# The immunobiology of the mammalian epididymis: the black box is now open!

**DOI:** 10.1186/2051-4190-23-8

**Published:** 2013-10-02

**Authors:** Rachel Guiton, Joelle Henry-Berger, Joël R Drevet

**Affiliations:** GReD Laboratory, CNRS UMR 6293 - INSERM U1103, Clermont Université, Clermont-Ferrand, France

**Keywords:** Spermatozoa, Immune control, Inflammation, Peripheral tolerance

## Abstract

Spermatozoa represent an immunologic challenge for the mammalian males. They are produced long after the establishment of the immune library of the individual and harbor specific spermatic antigens that are found nowhere else in other organs, tissues and cells. Consequently, spermatozoa are somehow “foreign” to the male adaptive immune system. In order not to elicit autoimmune responses that would be detrimental for male fertility, spermatozoa should be either physically separated from the adaptive immune response and/or, the immune system challenged by spermatic antigens must be efficiently silenced. Within the mammalian male genital tract it becomes more and more obvious that a range of strategies are at stake to ensure that the immune-stranger spermatozoa do not constitute an immunological issue. In this review the focus will be on the immune status of the epididymis tubule, in which spermatozoa that have left the testes will mature for approximately 2 weeks and may be stored for prolonged period of time. How the epididymal immune environment compares to that of the testis and what are the immune regulatory processes at work in the epididymal compartment will only be briefly described. Instead, this review will focus on recent data that highlight epididymal immune regulatory actors that partly explain/illustrate the rather complicated, fragile but nevertheless robust immune environment of the epididymis.

## Résumé

Les spermatozoïdes constituent un défi immunologique pour le mammifère mâle qui les fabrique. Elaborés longtemps après l’établissement de la librairie immunitaire de l’individu, ils portent des antigènes spécifiques de la lignée germinale qui leur confèrent un caractère “étranger” de nature à solliciter le système immunitaire. Afin de ne pas engager de réponses auto-immunes anti-spermatozoïde dont l’issue serait délétère pour la fertilité de l’individu, les spermatozoïdes doivent être gardés à l'abri des cellules immuno-compétentes, ou, si une réponse auto-immune anti-spermatozoïde est engagée, cette dernière doit être rendue silencieuse. Au sein du tractus génital mâle il apparaît de plus en plus clairement que différentes stratégies sont à l’œuvre afin d’empêcher que le spermatozoïde ne “challenge” le système immunitaire du mâle. Cette revue présente le statut immunologique de l’épididyme des mammifères dans lequel les spermatozoïdes qui ont quitté le testicule vont maturer pendant environ 2 semaines et être éventuellement stockés pour des périodes de temps variables. En quoi la situation immunologique de l’épididyme est-elle comparable ou différente de celle du testicule et quels sont les mécanismes immunitaires en action dans ce tubule seront présentés de façon non exhaustive. Une attention particulière sera donnée à des résultats récents qui mettent en avant quelques acteurs clefs de l’immuno-modulation et de la tolérance immune au sein de ce tissu épididymaire.

## Introduction

The mammalian immune system consists in a very elaborate network involving organs, tissues, cells and molecules that interact to determine what is self (and in good integrity) that should be ignored from what is non-self (or self that has gone awry) and should be eliminated. The distinction self/non-self is not always that clear-cut and there are situations where tolerance is necessary. One classical example is the tolerance that should be given to commensal bacteria populating the intestinal compartment.

In mammals, tolerance is established at two different levels and at two different periods. Central tolerance is set up in the primary lymphoid organs (bone marrow and thymus) essentially prior to birth by the absence of release of auto-reactive immune competent T and B cells. The second level of tolerance called peripheral tolerance is realized continuously in the tissues by means of various immunosuppressive activities and/or tissular organizational arrangements (see Table 1). The situation of immune privilege towards spermatozoa established in the mammalian male genital tract falls in that second category.

**Table 1:** **Comparison of central** ***versus*** **peripheral tolerance mechanisms**

	When?	Where?	How?
**Central tolerance**	Fetal life (setting)	Thymus (T lymphocytes)	Apoptotic death **(deletion)** of immature self antigen-specific lymphocytes
	Throughout life (maintenance)	Bone marrow (B lymphocytes)	
**Peripheral tolerance**	Throughout life	Whole organism (except thymus and bone marrow)	Apoptotic death **(deletion)** of mature self antigen-sepcific lymphocytes
			OR
			Functional inactivation **(anergy)**
			OR
			**Suppression** of lymphocyte activation and functions by regulatory T cells (Tregs) or by other immunosuppressive effectors such as IDO, kynurenines, cytokines (IL-10), growth factors (TGF-β1)…

Central tolerance is a very early mechanism that is maintained throughout life. It takes place in the primary lymphoid organs (thymus and bone marrow) and consists in the deletion of self antigen-specific B and T cells as soon as they are produced. Peripheral tolerance is set up after central tolerance and is maintained in the whole organism throughout life. Three mechanisms can act: deletion and anergy of mature self antigen-specific lymphocytes, and effector T cell (Th17, Th1, Th2) suppression by regulatory T cells (Tregs) or by immunosuppressive molecules (indoleamine 2,3-dioxygenase = IDO, kynurenines, IL-10, TGF-β1…).

Although the biological challenge is the same (to avoid autoimmune responses against sperm antigens), the different tissues of the male genital tract do not solve it the same way. As for many other physiological aspects, the testis is the site of the male genital tract where the immune situation has been most studied these last years and where we have a quite clear view of the innate and adaptive immune mechanisms at work. Since the present review focuses on the immune environment of the epididymis tubule it will not cover in detail the situation in the testis. Recent extensive reviews are however available for interested readers [1–3]. Briefly, the first major factor restricting the engagement of immune responses towards spermatozoa in the seminiferous tubules is the tight organization of the epithelium. The high efficiency of the blood-testis-barrier (BTB) isolates the luminal compartment of the seminiferous tubule from the interstitial compartment surveyed by immune cells [4]. The seminiferous epithelium is therefore particularly protected from the passage of immune cells and immune regulatory molecules because of the specialized tight junctions that lock Sertoli cells to one another. In addition, these isolating junctions are found at the basal-lateral membrane domain, a situation that is not that of classical mucosal epithelia and in particular not that of the epididymis epithelium. In that respect the immune situation of the epididymis tubule resembles more that of the intestine epithelium with the difference that, up to now, no one has found in the epididymis tubule specialized mucosa-associated lymphoid formations such as the Gut-Associated Lymphoid Tissue (GALT) in which the adaptive immune response is partly orchestrated.

### The immune context of the epididymal epithelium

Clinical observations support the idea that the mammalian epididymis immune control is rather different from that of the testis. Firstly, epididymitis is largely more frequent than orchitis and the latter very often leaks to epididymo-orchitis while the reverse is not that frequent. Secondly, acute epididymitis is essentially provoked by retrograde invasion of urethral bacterial pathogens in sexually transmitted disease (STD) situations, while orchitis is more frequently due to blood-transmitted pathogens (for a recent review see: [3]). This suggests that despite their luminal connection through the efferent ducts, the immune regulatory mechanisms that control both the seminiferous and epididymal tubules are probably not the same and that the proximal part of the epididymis (*ie* the *caput*) may function as a control point limiting the proliferation of ascending pathogens. Third, the blood-epididymis barrier (BEB) (for a recent report on the mammalian BEB see: [5]) appears to be much weaker compared to the BTB since it is quite frequent to observe intra-epithelial immune cells as well as luminal leucocyte infiltrations in the epididymis when compared to the seminiferous tubules [3]. Immunoglobulins too have been shown to access the epididymal luminal compartment [3]. In addition, within the different cell types that constitute the epididymis epithelium it is yet not clear whether or not some of these cell subtypes have immunological functions. This is particularly the case of the so-called halo and basal cells, the latter cell type having been shown to express macrophage-specific markers, at least in the mouse model [3].

#### Antigen presenting cells (APC) in the epididymis epithelium

Interestingly, it was recently shown that the mouse epididymis epithelium exhibits a dense network of dendritic-like cells [6] with a stellate morphology and cytoplasmic projections that may reach the luminal compartment and, consequently, be in a position to sample its content. This finding somehow changes our vision of the epididymis tubule that, up to now, was considered as a territory devoid of antigen presentation events. It brings forward the idea that the epididymis is active in maintaining self-tolerance towards spermatozoa and that it is not solely under the protective influence of the peripheral tolerance established in the testis, as it was often suggested earlier. Again, this observation reinforces the idea that the epididymis tubule resembles the intestinal situation where dendritic cells were shown to play an important role through their ability to orchestrate protective immunity and immune tolerance in the host [7].

The epididymal dendritic-like cells (eDCs) were shown to be particularly dense in the proximal *caput* epididymidis where the dendrites seem to protrude beyond the apically located tight junctions constituting the BEB. Further down the epididymis tubule, the eDCs were found less numerous and their dendrites less invasive within the epididymal epithelium. In the *cauda* epididymidis, the eDCs were shown to have no dendrites and to be restricted to the basal compartment of the epithelium [6]. These characteristics of distribution suggest that the eDCs are in a position of sampling the luminal content of the proximal epididymis, but not further down the epididymis tubule.

In recent years, dendritic cells (DCs) have received a lot of attention and are now thought to have a key role in bridging innate immunity with the induction of adaptive immunity [8]. In addition, as previously mentioned, DCs have been shown to orchestrate both central tolerance and peripheral tolerance [8]. Several categories of DCs were described starting with classical DCs (cDCs), which exhibit a strong antigen-presentation capacity and therefore are major T-cell inducers. In relation with their high ability to present antigens cDCs are characterized by their high expression of MHC class II antigens and integrin CD11c on their surface. It is interesting to note that these features were found on the epididymal DCs [6]. In addition, Da Silva *et al.* reported that, at least *in vitro*, the epididymal DCs have strong antigen-presenting abilities [6] reinforcing the idea that they are of the cDC type. The *in vivo* activity of the epididymal DCs was recently further illustrated by the recent report from the same group that after efferent duct ligation (known to provoke apoptosis in the epididymal proximal epithelium), epididymal DCs engulf and evacuate apoptotic cells to maintain the integrity of the epididymis tubule (Tegan Smith, *selected oral communication at the 2013 International Congress of Andrology, Melbourne Australia*). While doing so, it was observed that the eDCs dendrites retract. These behaviors at least illustrate the phagocytic capacity of the eDCs in the very proximal segment of the epididymis.

The second well-characterized group of DCs is the plasmacytoid DCs (pDCs) that are essentially found in the circulation and in the peripheral lymphoid tissues. These pDCs are not good antigen-presenting cells and consequently they express low levels of MHC class II antigens as well as the co-stimulatory molecules important for T-cell activation. Upon activation pDCs have been shown to secrete large amounts of IFN-α and IFN-β, which suggests that they play an important role in anti-viral immune responses [9]. Whether this subtype of DCs exists in the epididymis is not known because in mouse, in contrast to the human situation, pDCs also express at their surface the CD11c marker. Therefore, Da Silva *et al.* in their pioneering study [6] could not discriminate between cDC and pDC in the epididymis.

Not all DCs are stimulatory DCs since it was shown that regulatory DCs also exist in various settings. At the beginning it was thought that immature DCs could induce immunosuppression and tolerance [10]. However, it was recently shown in different tissues and situations that mature tolerogenic DCs do exist. Of note is the report that gut DCs expressing the CD103 marker were strongly involved in inducing tolerance through their ability to promote the differentiation of immunosuppressive T-regulatory cells from naïve T-cells (the so-called T-regs secreting the immunosuppressive effectors TGF-β and IL-10) [11]. The capacity of these gut CD11c^+^CD103^+^ DCs to induce T-reg cells was further associated with their ability to express the immunosuppressive enzyme indoleamine 2,3-dioxygenase (IDO: EC 1.13.11.42) ([12, 13] and see below). Da Silva *et al.* have shown that at least part of the epididymal CD11c^+^ DCs they have brought forward in the epididymis epithelium were also positive for CD103 which strongly supports the idea that, similarly to the gut situation, tolerogenic DCs are present [6]. Rather intriguing was the observation (by Da Silva’s group and ourselves) that despite the fact that *caput* epididymal DCs were shown to share common markers with intestinal tolerogenic DCs (since both were CD11c^+^ and CD103^+^[6]) they were not found, at least by immunocytochemical approaches, to express the immunosuppressive effector indoleamine 2,3-dioxygenase that partly explains the tolerogenic action of DCs in the gut [14] as well as in other settings [13]. This observation was quite puzzling since IDO activity has long been known to be high in the mammalian epididymis [15]. The next chapter will focus on that particular immunomodulatory molecule that was recently investigated further in the mouse epididymis.

In summary, at least two populations of DCs are present in the epithelium and interstitial compartments of the mouse epididymis, classical immune response-activating DCs and tolerogenic DCs. How these different eDCs are distributed along the epididymis tubule remains to be shown both in normal and infected situations. The fact that the *caput* segments show more eDCs having a stellate/dendriform morphology compared to the *cauda* territory, is in agreement with the idea that tolerance towards sperm antigens should be at first efficiently maintained in the proximal part of the organ when spermatozoa enter the epididymis tubule. In the meantime the *caput* luminal compartment is also a territory that should be efficiently surveyed both for abnormal sperm cells and non-self ascending pathogens. For the latter, this survey is particularly important in order to protect the testis from the retrograde invasion of sexually transmitted pathogens. The eDCs, by efficiently sampling the *caput* luminal content, are likely to participate to these phenomena. It would be interesting to test whether in epididymal ascending infectious situations the *cauda* eDCs are activated or not. The DCs situation in the epididymis fits rather well the concept of “division of labor” within dentritic cell subsets which proposes that different subsets of DCs are at work to modulate the fine equilibrium between immune survey and tolerogenesis [8–10]. On these grounds, we speculate that luminal antigen presentation within that proximal tubule is probably quite active with “immunogenic” or also called “presenting” or else “stimulatory” eDCs sampling efficiently the epididymal luminal compartment. Efficient immune monitoring could also explain why this epididymal territory is so rarely concerned by epithelial tumorigenic events since the appearance of “foreign” tumor antigens would rapidly engage the immune system and the elimination of the tumorigenic cells. In the meantime, outside abnormal situations (such as infections and tumors) tolerogenic/regulatory epididymal DCs would ensure that the immune response towards spermatic antigens remains low. In addition, in that context of efficient antigen presentation in the proximal epididymis tolerance towards sperm antigens could also be established through the priming of particular subsets of T-cells that have immunosuppressive roles.

#### Epididymal-specific expression of immunomodulatory effectors

##### Indoleamine 2,3-dioxygenase and its downstream kynurenine metabolites

Indoleamine 2,3-dioxygenase is the first and rate-limiting enzyme in tryptophan catabolism through the kynurenine pathway [16]. It is a ubiquitously expressed cytoplasmic protein activated by interferons (IFNs). In recent years IDO has received a lot of attention and there is a large body of data showing that IDO plays key immunomodulatory roles in classical immune responses but also in particular situations such as fetal tolerance, tumor immune resistance, and regulation of autoimmune responses [17].

IDO was associated with the epididymis as early as the eighties [15] when it was shown that IDO activity was surprisingly high in epididymis extracts. Intriguingly, IDO expression in the epididymis was found to be constitutive and independent of its classical inflammatory cytokine inducer (IFN-γ) since IDO expression was identical when WT mice were compared to mice deficient for IFN-γ signaling [18]. This observation suggested that the epididymis is in a particular state of anti-inflammatory response since IDO is classically considered a component of early response to inflammation and infection. Besides knowing the immunomodulatory function attributed to IDO especially in down-regulating the adaptive T cell-dependent immune response, it was speculated that the intense epididymal expression of IDO was likely to participate in the establishment of an immunotolerant environment towards immune-challenging sperm antigens. In recent studies we have investigated further the expression and the roles of IDO in the mammalian epididymis using both WT and IDO1-deficient mouse models [19, 20]. We have shown that IDO1, but not IDO2 and TDO that belong to the same family and have the same roles, is preferentially expressed in the mouse epididymal epithelium. The minor roles played by the low expression of IDO2 and TDO in the epididymis was confirmed by the fact that when IDO1 expression was knocked-out (*ie* in the IDO1-deficient model) the level of kynurenines, the products of IDO activity, dramatically decreased. This suggests that in the absence of IDO1 there is no compensation by the other members of the family [19]. In addition, TDO was not found in the same cell types since it was localized in the smooth muscle cells lining the epididymal duct as well as in transiting spermatozoa [21].

IDO1 expression in the epididymis is regionalized since it was shown to be *caput*-restricted. Within the *caput,* IDO1 expression starts posterior to segment 1 and peaks up at segments 3 and 4. It then goes down in segment 5 to undetectable levels further down the epididymis tubule epithelium [21–23]. At the cellular level, IDO1 expression was shown to concern preferentially principal and apical cells within the epididymal epithelium [21, 22]. As said above *via* immunohistochemical approaches it was not found to localize in the eDCs where we logically thought it could be with reference to the gut situation where IDO expression is a feature of the CD11c^+^CD103^+^ DCs [14]. This observation suggests that the principal/apical epididymal cells somehow cooperate with the immune cells to regulate the immune environment. This is not a completely new situation since it was shown elsewhere that lung epithelial cells might contribute to protective tolerance *via* a (TLR3/TRIF)-dependent pathway converging on IDO [24].

We have very recently shown that when IDO1 is not present, *ie* in the *Ido1*^*−/−*^ mouse model, it drives the *caput* epididymidis in a more pronounced inflammatory state as evidenced by the significant increase of several inflammatory cytokines and chemokines, the induction of cyclo-oxygenases COX1 and COX2 and their associated lipid intermediates [20]. Nevertheless, despite the slightly more inflamed situation, the immune status of the tissue remains under control since we never observed acute inflammatory events such as the occurrence of granuloma. This suggests that some mechanisms are switched on to back-up the missing IDO activity. We also have shown in the same study that the absence of IDO1 expression and of its downstream kynurenine metabolites modifies slightly the epididymal representation in the various T cell subsets as it is logically expected. IDO is known to be a strong inducer of the immunosuppressive regulatory T-reg cell lineage at the expense of the Th17 inflammatory subset [25]. Thus, quite logically, in its absence we have shown that the differentiation of the Th17 T cell subset from naïve T cells is promoted [20]. Consequently, we also demonstrated that the stimulation of the Th17 inflammatory cells influences the Th1/Th2 equilibrium in favor of the Th1 sub-lineage [20]. This was confirmed by the cytokine profile since the Th1 inflammatory cytokines (TNF-α, IFN-γ, IL-1β and IL-6) were all significantly increased in *Ido1*^*−/−*^*caput* extracts while the typical Th2 cytokine IL-4 was not [20]. These observations were in agreement with the conventional roles of IDO and of its downstream kynurenine metabolites on immune reponses. Promotion of a Th1-driven response in the epididymis of *Ido1*^*−/−*^ animals was to be expected in the context of a weaker immunotolerant environment against sperm antigens that should stimulate a Th1-mediated autoimmune reaction [26]. In addition, it has been reported that kynurenines including 3-hydroxy-arachidonic acid (3-OH-AA) and 3-hydroxykynurenine (3-OHK) inhibit the actions of Th1 cells and enhance the action of Th2 cells [27]. Other reports have also shown that stimulation of IDO activity in dendritic cells diminishes Th1 response [28]. Conversely, it was also reported that 1-methyltryptophan-mediated inhibition of IDO activity enhances Th1-associated inflammation [29]. Therefore, in the absence of IDO1 and of its downstream metabolites, as it is the case in the epididymis of the *Ido1*^*−/−*^ animals [7], a shift of the Th1/Th2 equilibrium towards Th1 was to be expected.

It was also quite interesting to note that the absence of IDO1 expression in the epididymis was associated with a weaker capacity of the epididymis to realize an efficient quality control function on transiting sperm cells. This was evidenced by the fact that in the *Ido1*^*−/−*^ model we recorded in the *cauda* storage compartment twice the number of spermatozoa as in control WT animals. This was neither associated with a difference in the efficiency of spermatogenesis nor with a difference in sexual activity [20]. The greater sperm counts found in *Ido1*^*−/−*^*cauda* epididymides were essentially due to an increase in spermatozoa having abnormal morphology as well as necrotic spermatozoa that, in a normal context, should have been disposed of during epididymal descent [19]. These observations reinforce the rather controversial idea that sperm selection during epididymal descent is a process that is partly under the control of the immune response [30]. To support the involvement of immune cells and immune responses in the epididymal selection of spermatozoa is our observation that the increased defective sperm counts found in *Ido1*^*−/−*^*cauda* epididymides were paralleled with a very significant decrease in the *cauda* fluid leucocyte content [19].

##### Cyclo-oxygenases, Cox2 and Cox1

It is worth noting that the epididymis is rather unusual as it is characterized by its constitutive high expression of COX2 [31, 32]. In any other tissue, COX2 expression is solely induced by inflammatory stimuli. The constitutive expression of both COX2 and IDO1 recorded in the mouse *caput* epididymidis reinforces the idea that this territory is in a permanent semi-inflammatory state characteristic of immunotolerant settings. We have shown that both COX1 and COX2 expression levels were up-regulated in *Ido1*^*−/−*^*caput* extracts compared to WT animals [20]. Although it is commonly believed that the constitutive isoform COX1 has little or no involvement in regulating immune responses [33], there are recent reports suggesting that COX1 is actively involved in immunoregulation [34] and that part of its effect is mediated *via* IL-17 production by Th17 cells [35]. Our data are consistent with these statements since we observed in the *caput* epididymides of *Ido1*^*−/−*^ animals a shift in the Th17/Treg equilibrium towards Th17 cells and an associated increase in the concentration of IL-17 [20].

##### Factors of the TGF-β family

The immunosuppressive functions of TGF-β are well recognized and documented. There are two ways by which TGF-β suppresses immune responses either through the inhibition of inflammatory cell functions or *via* the promotion of T-reg cell functions (for a recent review see [36]). It is interesting to note that in the slightly more inflamed *IdoI*^*−/−*^ epididymis we recorded an increase in the activation of the Smad3 intracellular effector of the TGF-β pathway [20]. It is possible that this TGF-β signal constitutes part of the mechanisms by which the immunosuppressive response of the epididymal tissue is maintained in the absence of IDO1 and kynurenines. It may also explain why in the absence of IDO and kynurenines we do not lose the T-reg subset. Indeed, TGF-β (more specifically TGF-β1) was shown to act as a T-reg autocrine and paracrine inducer [37]. Which cell type(s) in the epididymis epithelium of *Ido1*^*−/−*^ animals is (are) involved in that surge of TGF-β signaling remains to be clarified. It is already known that the mammalian as well as the primate epididymides express significant levels of the different TGF-β isoforms in a region-specific manner [38–41]. More precisely, in the marmoset monkey, TGF-β1 was found expressed in epididymal apical epithelial cells while its receptor was found on the adjacent principal cells suggesting that paracrine TGF-β1 signaling could occur within the epididymal epithelium [39].

It is also worth noting that *via* comparative gene expression array analyses we revealed *Bmp8a* to be among the genes most strongly induced in *Ido1*^*−/−*^*caput* epididymides. BMP8a belongs to the TGF-β family and was previously shown to participate in the differentiation of the caput epididymal epithelium [42, 43]. Interestingly, lack of BMP8a and BMP7 expression in *Bmp8a*^*−/−*^ and *Bmp8a/Bmp7*^*−/−*^ mice provoked inflammatory situations in the *caput* epididymides of these animals characterized by the occurrence of sperm-mediated granulomas [43]. These observations support the idea that a TGF-β family member such as BMP8a might participate in the immune control of the adult *caput* epididymidis. It is thus possible that through an anti-inflammatory autocrine/paracrine signaling cascade involving the TGF-β/BMP/activin pathway the *Ido1*^*−/−*^ epididymal epithelium partly recovers its immune balance by maintaining the T-reg population and by negatively controlling the expansion of the Th17 sub-lineage.

##### IL-10

IL-10 is an immunosuppressive cytokine which along with TGF-β1 constitutes the signature of the T-reg lineage and suppresses the Th1/Th2/Th17-mediated immune response. We observed a significant increase in IL-10 production in the *Ido1*^*−/−*^*caput* extracts that may also participate in the new immune equilibrium reached by the tissue. Although T-reg cells are the major provider of IL-10, it has been shown lately that several other cell types can also produce it; including Th1, Th2, cytotoxic T cells, B lymphocytes, mast cells, mononuclear phagocytes, APCs as well as T^DN^ cells [44, 45]. More investigations will be necessary to identify which cell(s) type(s) in the *caput* epididymidis of *Ido1*^*−/−*^ animals is (are) responsible for the increased production of IL-10. In this respect, it is of interest to note that the principal cells of the epididymis epithelium have been shown earlier to express IL-10 and to have the capability to over-express IL-10 in some abnormal pathologic situations [46]. If this is verified, epididymal epithelial IL-10 expression could be yet another way by which autoantigenic spermatozoa are protected from immune destruction.

##### Retinoic acid/retinol

Recently immunologists started to explore the mechanisms by which vitamin A controls the innate and adaptive immune systems. It was shown that certain intestinal APC subsets including DCs metabolize vitamin A into retinoic acid (RA) and enhance the TGF-β-dependent conversion of naïve T cells into regulatory T cells (for a recent review see [11]). In addition, RA synthesis by APC was shown to influence the migration of T and B cells as well as IgA into mucosal sites. Beside DCs, non-hematopoietic cells of the gut such as epithelial and stromal cells have been shown to also synthetize RA and participate in these migratory and homing processes of immune cells and immunoglobulins. Therefore, epithelial cells once again somehow cooperate with APCs to control local immune responses in both inflammation and tolerance (for a recent review see [47]). In this context, it is interesting to remember that RA metabolites have long been shown to play an important role in the mammalian epididymis [48, 49]. In situation of vitamin A deficiency as is the case in transgenic animals expressing a dominant negative form of retinoic acid receptor α it was reported that the *cauda* epididymidis is dysfunctional leading to a situation of infertility [50]. Thus, trafficking of retinoids is likely to be a regulator of epididymal function. Knowing the recent roles devoted to RA metabolites in modulating immune responses it would not be surprising to find out that RA participates in the immune equilibrium of the epididymis. One interesting point is the rather recent observations bringing forward the interplay between RA and TGF-β signaling in inducing T-reg differentiation [51].

#### Presence of peculiar lymphoid cell sub-lineages

In the epididymis, in contrast to the testis, both intra-epithelial and interstitial immune cells are rather frequent (for a recent review see [2]). Regarding lymphocytes, the prevalent view is that overall they are more frequent in the proximal epididymis (*caput)* compared to the distal regions. Within the organ itself, it was reported that the interstitium preferentially contains CD4^+^ T cells (of the assistant and regulatory types, Ths and T-reg) while the epididymal epithelium shows more CD8^+^ T cells (of the cytotoxic type, Tc) (reviewed in [2]). Our recent investigations, reported in [20] have used FACS analysis to look at the distribution of leucocytes in *caput* epididymidis tissue samples dissociated by collagenase treatment in both WT and *Ido1*^*−/−*^ animals. In total agreement with previous data, our analyses revealed that B cells and CD3^+^ T cells were rather rare in the *caput* epididymal tissue and that most of the lymphoid cells were triple negative (CD3^-^CD4^-^CD8^-^) cells so most likely macrophages, DCs or/and NK cells [52]. Regarding the few CD3^+^ cells, they were essentially found to be of the CD3^+^ double negative type (devoid of CD4 and CD8), the so-called CD3^+DN^ or T^DN^. Recently, T^DNs^ have been added to the increasing list of T cells that exert regulatory functions similar to those of classical T-reg (CD4^+^CD25^+^FoxP3^+^) cells [53–57]. Interestingly, T^DNs^ were recently shown to be involved in the prevention of autoimmune responses engaged in the inflamed epididymis after vasectomy [58]. In agreement with the observation that the epididymal immune equilibrium of the *Ido1*^*−/−*^ animals is somehow well preserved we have not seen any significant change in the representation of the various leucocyte lineages in the *caput* epididymides of this genotype.

#### Immune assistance of the epididymal white adipose tissue

In rodents, the epididymis is surrounded by a specific white adipose tissue (WAT) called the epididymal WAT or EWAT. It was interesting to note that one of the associated phenotypes of the *Ido1*^*−/−*^ animals was a significant reduction of the volume of the EWAT at 6 months of age which was confirmed and even accentuated in animals aged 12 months, compared to WT animals at the same ages (JHB & RG, unpublished data). It is tempting to propose that the proximal EWAT somehow participates in the mechanisms that regulate the inflammatory status of the *Ido1*^*−/−*^*caput* epididymidis. At this stage of our investigations, it however cannot be excluded that the proximal EWAT loss is just a collateral effect of the *caput* inflammation. How could the EWAT participate in the immune control of the epididymis? It is well established that the WAT constitutes an immune and inflammatory cell reservoir [59] and that each adipose depot presents, according to its localization, different immunological properties [60]. In that respect, it is interesting to note that the EWAT is highly enriched in anti-tumoral NK and NKT cells, characteristics shared with epithelial tissues where these immune cells help in maintaining tissue integrity, antitumoral surveillance, defense against pathogens and in regulating inflammation [61, 62]. Murine adipose stem cells (mASCs) were recently shown to down-regulate both Th1-driven autoimmune and inflammatory responses in Crohn’s disease (a chronic disease characterized by severe Th1 cell-driven inflammation of the colon) [63]. In that particular report, mASCs were shown to decrease a wide panel of inflammatory cytokines and chemokines, to increase interleukin-10 levels, to impair Th1 cell expansion, and to induce/activate both *in vitro* and *in vivo* CD4^+^CD25^+^FoxP3^+^ regulatory T cells with a suppressive impact on Th1 effector responses. In the light of these observations, it is possible that the EWAT ASCs play such an immunomodulatory role in the *Ido1*^*−/−*^ animals at the expense of their local adipogenic function. Concurring with Chu *et al.* (2010) [64], who speculated that the EWAT pad might have a facilitative/supportive role in the production and the maturation of sperm *via* the local and direct action of unknown EWAT-produced factors, we propose to extend this supportive local role of the EWAT to the maintenance of the immune balance in the *caput* epididymidis. However, it should be remembered that this situation is particular to rodents since there is no mention of such adipose depots in larger mammals, including human.

#### The particularities of the epididymal initial segment of the caput

Once again the *caput* segment 1 or initial segment of the epididymis is distinct. Unlike the rest of the tubule, the initial segment is highly vascularized and presents a dense network of capillaries irrigating the entire surface of the segment. This is responsible for its pink appearance easily visible upon dissection of the tissue when the conjonctiva is removed. This segment is also rather more heavily drained by lymphatic vessels than the rest of the tubule ([65] and JHB & RG, unpublished data). The well-developed lymphatic drainage of the *caput* epididymidis distinguishes it from other tissues, such as the brain in which an immune privileged situation is associated with absence of lymphatic drainage. Therefore, in this segment 1, if one associates the presence of conventional presenting dendritic cells with a dense lymphatic system it is likely that what is taken up and processed by the eDCs will end up in the nearest local lymph nodes where presentation to immune competent cells should take place. How the immunogenic and tolerogenic responses are then orchestrated in this territory will have to be investigated. Most likely, the concept of division of labor within the different subsets of APCs will explain the situation. For example, outside infectious events classical eDCs may target T regulatory cells while upon pathogen-mediated inflammatory situations, inflammatory eDCs could dialog with effector T cells. Both the cytokine environment and the type of mature eDCs could then drive the immune equilibrium response from a tolerant state towards an immunogenic state. At this stage, we view the initial segments of the epididymis as a very important checkpoint of the tubule where an extremely fine tuning of immune mechanisms exists to serve three major goals: 1) maintenance of tolerance towards sperm antigens, 2) last watch for ascending pathogens that could invade the high security gonad quarter, 3) elimination of abnormal spermatozoa.

## Conclusions

**Figure 1:**
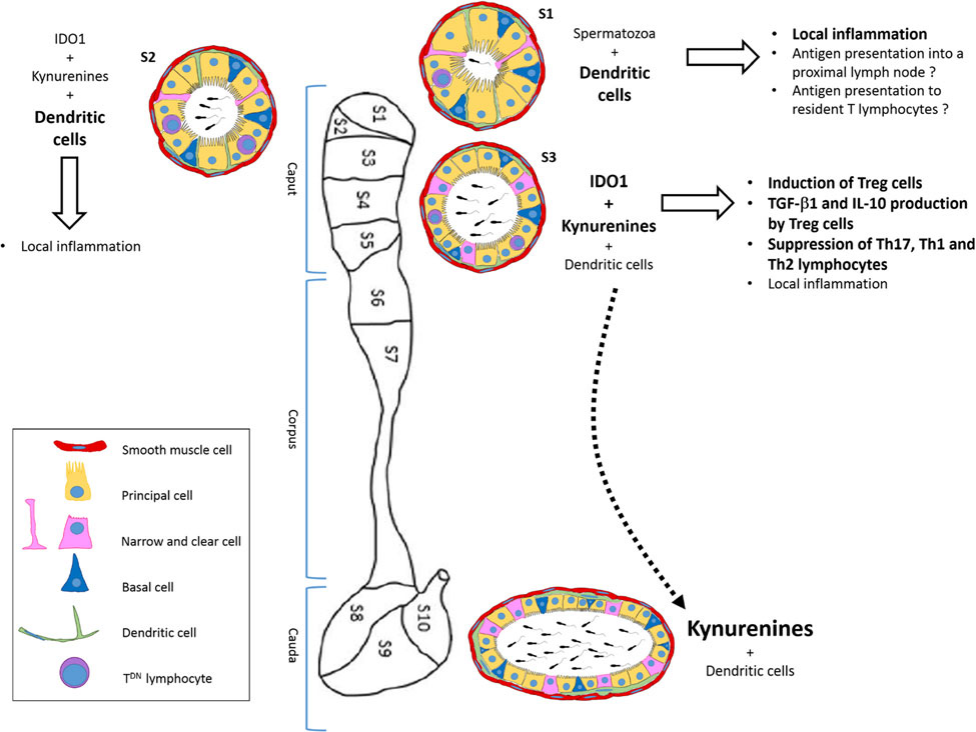
**Hypothesis of the setting and maintenance of immune tolerance to spermatozoa in the epididymis.** It seems that various mechanisms/cells/molecules are at work in the different epididymis segments. The first segment (S1) is filled with a dense network of dendritic cells able to reach the lumen. They could thus potentially sample luminal antigens (and among them sperm antigens) to present them to local (epithelium-resident T cells) or distant (proximal lymph node-resident T cells) immunoregulatory cells. The lack of immunomodulatory molecules could explain the slight inflammation we previously described in this segment. Spermatozoa entry into the epididymal tubule induces the expression of indoleamine 2,3-dioxygenase (IDO1), which can be found as soon as the second segment (S2), and of its enzymatic products, the kynurenines. The combination of numerous dendritic cells and IDO1 expression leads to a reduction in the local inflammation observed in the initial segment. IDO1 expression reaches its maximum in the third segment (S3) leading to a strong increase in kynurenine concentrations. The later induce the differentiation of regulatory T lymphocytes which produce immunomodulatory molecules such as TGF-β1 or IL-10, ending in the suppression of effector T cells (Th17 or Th1). In the same time, the loss of dendrites on the dendritic cells suggests that at this level of the epididymis tubule they may become accessory cells in the maintenance of tolerance.

Recent investigations have started to open the black box of the mammalian epididymal immune physiology. The initial hypotheses that epididymal luminal antigen presentation was absent and that solely innate phenomena were in charge of the immune watch of this territory are now challenged by the evidence that professional antigen presenting cells such as dendritic cells (DCs) are present throughout the tissue. Because recent advances ascribe various subsets of DCs fundamental roles in connecting innate immunity with adaptive immunity and in maintaining the balance between immunity and tolerance, as it is the case in the intestine, it is likely that the epididymal DCs will prove to play a crucial role in the immune equilibrium of the epididymis. Apparently, together with these classical immune cells, the epididymal epithelial cells also participate in the immune control of the organ. Besides the well documented ability of the epididymis epithelium to synthesize and secrete antimicrobicidal molecules which were not the topic of this review, the strong epithelial expression of the immunomodulatory IDO1 protein is to date the best illustration of this epithelial assistance in the control of the immune adaptive response. TGF-β signaling, IL-10 production, retinoic acid metabolism, the presence of peculiar T cell subsets such as the regulatory T-reg and T^DN^ may represent other ways by which the epididymal epithelium contributes to maintain the rather complicated immune situation of the tissue (summarized in Figure 1). Local crosstalk with the surrounding white adipose depot might also represent one side of the immune environment of the epididymis, especially in the *caput* region. The situation may appear complex but one has to remember that nowhere else the immune system has to face such a challenging act. Robust protection against invading ascending pathogens, immune tolerance towards immunogenic spermatic antigens and controlled autoimmune processes to ensure sperm selection and quality control are the three facets of the epididymis immunologic equation. It is obvious that we are still far from understanding the rather finely tuned immune context of the epididymis that could be translated into clinical applications. More investigations are definitely necessary. It is however unfortunate that very few research groups are presently involved in such research. Understanding the fine tuning of immune responses in the epididymis has obvious clinical relevance to the field of mammalian fertility. It could bring insights that reach far beyond reproductive issues since as said above the peculiar immune monitoring of the epididymis may well explain why this epithelium is so refractory to carcinogenesis which by nature represents an immunologic failure.

## Authors’ information

Dr. Rachel Guiton is Assistant-Professor at Blaise Pascal University-Clermont2, France. She has been trained in immunology at the Center of Immunology at Marseille-Luminy (CIML), France, and has an expertise on dendritic cell physiology in various settings. She was hired at the GReD laboratory in September 2012 and joined the MEPTI = (Mechanisms of Post-Testicular Infertility)’s research group. She is now involded in understanding the innate and adaptive immune responses of the mammalian epididymis.

Dr. Joelle Henry-Berger is a member of the GReD MEPTI’s research team who has contributed extensively to the production of the data reported on the *Ido1*^*−/−*^ mouse model.

Professor Joël R. Drevet, is the leader of the MEPTI’s (Mechanisms of Post-Testicular Infertility) research group and the adjunct-director of the GReD Laboratory (CNRS Unit UMR 6293-INSERM Unit U1103-Clermont Université) at Blaise Pascal University-Clermont2. Prof. Drevet has editorial duties for *Human Reproduction, PLoS ONE, Andrology, Asian Journal of Andrology, ISRN Urology* and *Basic & Clinical Andrology*. He seats at the board of the French Andrology Society (SALF) and is affiliated to the European Academy of Andrology (EAA), the International Society of Andrology (ISA) and the Society for Study on Reproduction (SSR).

## Abbreviations

BMP8a: *Bone morphogenetic protein 8a*
CD11c: Cluster of differentiation antigen 11c
CD103: *Cluster of differentiation antigen 103*
IDO1: *Indoleamine dioxygenase 1*
IDO2: *Indoleamine dioxygenase 2*
IFN-α: *Interferon alpha*
IFN-β: *Interferon beta*
IgA: *Immunoglobulin A*
IL-1β: *Interleukin 1 beta*
IL-4: *Interleukin 4*
IL-6: *Interleukin 6*
IL-10: *Interleukin 10*
IL-17: *Interleukin 17*
MHC class II: *Major histocompatibility complex antigens of class II.*
NK: *Natural killer*
NKT: *Natural killer-like T cell*
Smad3: *Similar to mother against decapentaplegic factor type 3*
TDN: *Double negative T cell*
TDO: *Tryptophan dioxygenase*
TGF-β: *Transforming growth factor-beta*
TLR3: *Toll-like receptor 3*
TRIF: *Toll-like receptor interacting factor*.
